# Rewiring yeast osmostress signalling through the MAPK network reveals essential and non-essential roles of Hog1 in osmoadaptation

**DOI:** 10.1038/srep04697

**Published:** 2014-04-15

**Authors:** Roja Babazadeh, Takako Furukawa, Stefan Hohmann, Kentaro Furukawa

**Affiliations:** 1 Department of Chemistry and Molecular Biology, University of Gothenburg, Gothenburg, Sweden

## Abstract

Mitogen-activated protein kinases (MAPKs) have a number of targets which they regulate at transcriptional and post-translational levels to mediate specific responses. The yeast Hog1 MAPK is essential for cell survival under hyperosmotic conditions and it plays multiple roles in gene expression, metabolic regulation, signal fidelity and cell cycle regulation. Here we describe essential and non-essential roles of Hog1 using engineered yeast cells in which osmoadaptation was reconstituted in a Hog1-independent manner. We rewired Hog1-dependent osmotic stress-induced gene expression under the control of Fus3/Kss1 MAPKs, which are activated upon osmostress via crosstalk in *hog1Δ* cells. This approach revealed that osmotic up-regulation of only two Hog1-dependent glycerol biosynthesis genes, *GPD1* and *GPP2*, is sufficient for successful osmoadaptation. Moreover, some of the previously described Hog1-dependent mechanisms appeared to be dispensable for osmoadaptation in the engineered cells. These results suggest that the number of essential MAPK functions may be significantly smaller than anticipated and that knockout approaches may lead to over-interpretation of phenotypic data.

All living cells respond to extracellular stimuli such as hormones, growth factors, cytokines, nutrients and stress. The information is processed by signal transduction systems, which mediate appropriate responses including altered gene expression, metabolism, secretion, proliferation and apoptosis. A conserved family of mitogen-activated protein kinases (MAPKs) serves major roles in intracellular signal transduction from yeasts to mammals^1^. MAPKs have numerous targets which they regulate at transcriptional and post-translational levels. In the budding yeast *Saccharomyces cerevisiae*, which has five distinct MAPKs (Hog1, Fus3, Kss1, Slt2/Mpk1, and Smk1)^2^, the osmoregulatory Hog1 MAPK (a mammalian p38 MAPK homologue) controls gene expression, glycerol accumulation, signal fidelity, and cell cycle arrest under hyperosmotic conditions^3^,^4^ (Fig. 1a). Consequently, deletion of *HOG1* confers osmosensitivity. Several hundred genes are upregulated upon osmotic stress and expression of about 50 of those genes is strongly dependent on Hog1^5^,^6^. Hog1 affects by phosphorylation the activity of numerous proteins, such as transcription factors^7^, cell cycle regulators^8^ and metabolic enzymes^9^. In this work, we investigated whether Hog1 requires all of those targets for mediating osmoadaptation. For this purpose, we chose a synthetic biological concept, i.e. reconstitution of osmoadaptation in the *hog1Δ* mutant.

Yeast is a highly attractive model organism for the study of MAPK signalling systems and for developing and testing synthetic biological approaches^10^. Engineering signalling pathways has significant potential to provide novel and complementary information that cannot be achieved by traditional genetic approaches such as gene knockout and overexpression^11^,^12^. For instance, rewiring signalling components between MAPK pathways^13^,^14^, introducing synthetic negative or positive feedback loops^15^,^16^, tethering signalling components with specific localization motifs^17^, assembling or recombining modular signalling domains^18^,^19^ and reconstitution of a heterologous MAPK cascade^20^ are highly informative for understanding the design principles of MAPK pathways and enable generating novel signalling properties. In the present study, we reconstituted osmoadaptation in *hog1Δ* cells by rewiring osmostress signalling through the MAPK network. This reconstitution approach revealed that osmotic induction of only two Hog1-dependent genes, which encode the enzymes required to produce the osmolyte glycerol, is sufficient for successful osmoadaptation. Moreover, analyses of yeast cells with synthetic osmoadaptation suggest that some of the well-known roles of Hog1 do not seem to be truly essential for osmoadaptation, at least not in the engineered cells. Hence, it appears that the number of MAPK functions essential for specific response may be significantly smaller than anticipated from knockout approaches and genome-wide analyses.

## Results

### Among Hog1-dependent osmostress-induced genes only *GPD1* is essential for osmoadaptation

**Figure 1:**
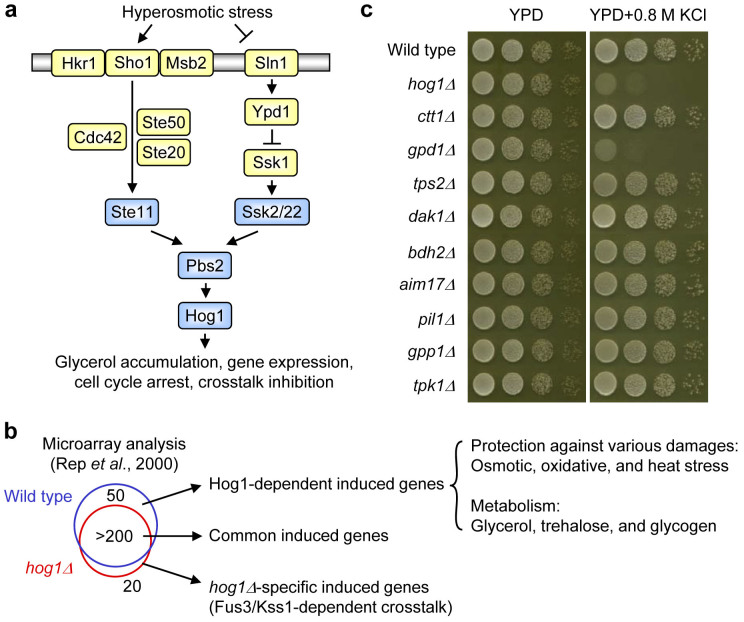
Among Hog1-dependent osmostress-induced genes only *GPD1* is essential for osmoadaptation. (a) Schematic diagram of the Hog1 MAPK pathway in *S. cerevisiae*. The HOG pathway consists of two upstream osmosensing branches (Sln1 and Sho1) each with a downstream MAPK cascade (Ssk2/Ssk22 and Ste11 MAPKKKs, Pbs2 MAPKK, and Hog1 MAPK). Activation of the HOG pathway leads to rapid translocation of Hog1 into the nucleus, which in turn stimulates expression of osmo-responsive genes via several transcription factors. In addition to gene expression, Hog1 plays roles in glycerol accumulation, control of cell cycle progression, cross pathway inhibition and other aspects of cell physiology. Upstream osmosensing systems are shown in yellow, and MAPK cascades in blue. (b) Hog1-dependent osmostress-induced genes identified by microarray analysis^6^ include genes encoding different stress protective proteins and metabolic enzymes. (c) Deletion of all strongly Hog1-dependent genes except *GPD1* does not cause an osmosensitive phenotype. Strains (see further mutants listed in Table S1) were grown on YPD plates with or without 0.8 M KCl for 1–2 days at 30°C.

We and others have previously analyzed the transcriptional response to osmotic shock in *Saccharomyces cerevisiae* and found that the mRNA level of 200 to 300 genes increased at least 3-fold upon stress^5^,^6^. About fifty of those induced genes were highly dependent on the presence of Hog1 and those Hog1-dependent genes encode proteins that presumably contribute to protection against different types of damage or encode enzymes in glycerol, trehalose, and glycogen metabolism (Fig. 1b and Table S1). To determine whether those gene products are required for osmoadaptation, we performed growth assay of the corresponding deletion mutants. Growth of the wild-type, *hog1Δ*, and 9 mutant strains chosen as examples (because they have been reported to show osmosensitive phenotype in the *Saccharomyces* genome database) are shown in Figure 1c. The rest of the mutant strains are shown in Table S1. Only the *gpd1Δ* strain (*GPD1* encodes glycerol-3-phosphate dehydrogenase, the first step in glycerol biosynthesis^21^,^22^) showed osmosensitivity similar to the *hog1Δ* mutant. None of the mutants lacking genes (e.g. *ald3Δ*, *ctt1Δ*, *hsp12Δ*, *stl1Δ*) whose transcriptional induction is stronger or more dependent on Hog1 than that of *GPD1* was osmosensitive. Hence, glycerol biosynthesis appears to be one of the most critical factors for Hog1-dependent osmoadaptation. Moreover, the fact that Hog1 mediates strong upregulation of many genes dispensable for osmoadaptation under laboratory conditions is consistent with a previous report showing no apparent correlation between gene expression and gene dispensability under osmostress conditions^23^.

### Synthetic osmoadaptation in *hog1Δ* cells using crosstalk between MAPK pathways

**Figure 2:**
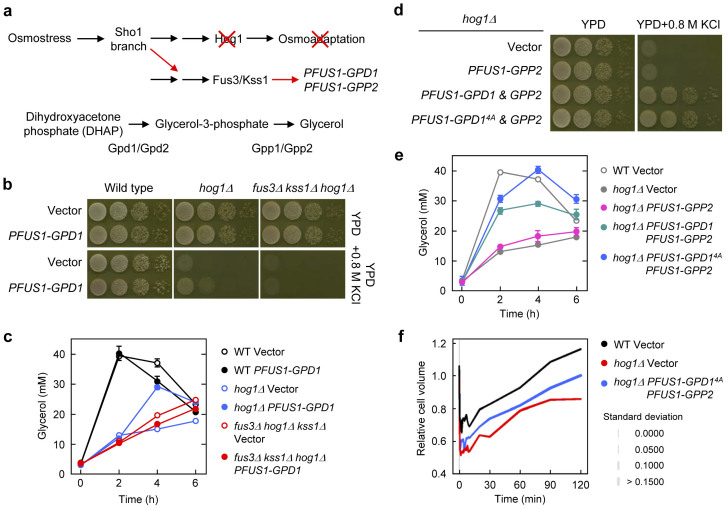
Synthetic osmoadaptation in *hog1Δ* cells. (a) Experimental design for synthetic osmoadaptation in *hog1Δ* using crosstalk and the glycerol biosynthesis pathway. Expression of Hog1-dependent osmostress-induced genes (*GPD1* or/and *GPP2*) was rewired under the control of a Fus3/Kss1 dependent *FUS1* promoter. (b) Osmotic induction of *GPD1* expression via crosstalk partially suppresses osmosensitivity of *hog1Δ* in a Fus3/Kss1-dependent manner. Cells of the indicated strains carrying YIp352 or YIp352-PFUS1-GPD1 were grown on YPD plates with or without 0.8 M KCl for 1–2 days at 30°C. (c) Intracellular glycerol accumulation correlates with the cell growth shown in (b). Cells were grown to mid-log phase, subjected to osmotic stress (0.8 M KCl), and intracellular glycerol was monitored at the indicated time points. Values represent the mean and standard deviation of three replicas. (d) Osmotic induction of *GPD1* and *GPP2* together via crosstalk strongly suppresses osmosensitivity of *hog1Δ*. Cells of the *hog1Δ* strains carrying YIp352-PFUS1-GPP2 and/or YIplac128-PFUS1-GPD1 (or GPD1^4A^) were grown as in (b). Cell growth shown in (d) correlates well with the ability of those cells to accumulate glycerol (e) and recover cell volume (f) after osmotic treatment. For cell volume data, line thickness indicates standard deviation of data obtained with approximately 30 cells. See Methods for the details of glycerol assay and cell volume measurement.

It is known that overexpression of *GPD1* with a multi copy plasmid partly suppresses the hyper-osmosensitive phenotype of *hog1Δ* cells^22^. On the basis of this fact and the result described above, we hypothesized that osmotic up-regulation of *GPD1* expression would suppress the phenotype of *hog1Δ*. We examined this hypothesis by reconstituting osmoadaptation in a Hog1-independent manner. We rewired osmostress signalling to the Fus3/Kss1 MAPKs, which is improperly activated via crosstalk in *hog1Δ* cells upon osmostress^6^,^24^,^25^. Specifically, we constructed a yeast strain in which *GPD1* is expressed under the control of the Fus3/Kss1-dependent *FUS1* promoter (Fig. 2a). As shown in Figure 2b, *hog1Δ* cells carrying a *PFUS1-GPD1* gene grew better than control cells on osmotic stress plates containing KCl (or NaCl and sorbitol: data not shown). Growth of the engineered cells (here called synthetic osmoadaptation) was dependent on the presence of Fus3/Kss1. Growth of these cells under osmotic stress (Fig. 2b) correlated well with the ability of those cells to accumulate glycerol (Fig. 2c). However, it should be noted that glycerol accumulation in the *hog1Δ* cells carrying *PFUS1-GPD1* started more slowly than in wild-type cells.

To make the *hog1Δ* cells adapt better to hyperosmotic stress, we examined another strongly Hog1-dependent gene, *GPP2*, which encodes glycerol-3-phosphate phosphatase^26^. Gpp2 catalyses the dephosphorylation of glycerol-3-phospate to glycerol, the second and final step in glycerol biosynthesis (Fig. 2a). In contrast to *GPD1*, osmotic induction of *GPP2* alone improved neither growth of *hog1Δ* cells on high osmolarity plates (Fig. 2d) nor glycerol accumulation (Fig. 2e). These results are consistent with a previous report showing that efficient glycerol production requires accumulation of glycerol-3-phosphate by Gpd1^27^. However, co-expression of *GPD1* and *GPP2* improved synthetic osmoadaptation. *hog1Δ* cells carrying both *PFUS1-GPP2* and *PFUS1-GPD1* genes grew under osmotic stress conditions and accumulated glycerol at a level close to wild type (Fig. 2d, e). Moreover, osmotic induction of an unphosphorylated form of *GPD1* (*GPD1^4A^*), which displays higher enzyme activity^28^, resulted in even better glycerol accumulation than that of wild-type *GPD1* (Fig. 2e). These results indicate that rapid and efficient glycerol accumulation suppresses osmosensitivity of *hog1Δ*.

Next, we determined whether the synthetic osmoadaptation can restore cell volume after osmotic shock by using a microfluidic device mounted under a fluorescence microscope^29^. The *hog1Δ* cells carrying *PFUS1-GPD1^4A^/PFUS1-GPP2* recovered cell volume more quickly than the control *hog1Δ* cells (Fig. 2f). This observation is consistent with recent reports showing that glycerol accumulation is essential for cell volume recovery^30^,^31^. Taken together, glycerol accumulation by upregulating expression of two genes, *GPD1* and *GPP2*, is sufficient for synthetic osmoadaptation.

### Hog1 is dispensable for regulation of Fps1 gating under hyperosmotic condition

**Figure 3:**
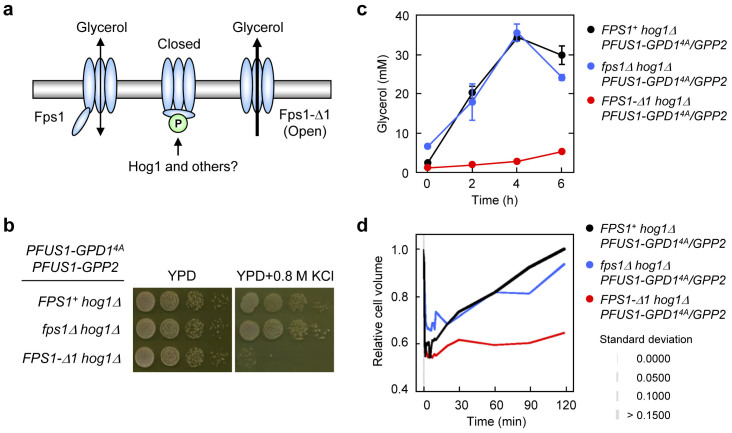
Hog1 is dispensable for regulation of Fps1 gating under hyperosmotic condition. (a) Schematic diagram of Fps1 regulation. Fps1 gating appears to be regulated by phosphorylation at Thr^231^ and eviction of its positive regulator Rgc2 (not shown), which are mediated by Hog1 and other kinases. Unregulated Fps1 (N-terminal truncated or Thr^231^ mutants) cannot close the gate and consequently causes osmosensitivity because of constitutive glycerol leakage. (b) The presence or absence of Fps1 does not affect synthetic osmoadaptation, while expression of Fps1-Δ1 prevents it. Cells of the *hog1Δ PFUS1-GPP2 PFUS1-GPD1^4A^* strains with vector (pRS403), *FPS1* deletion, or Fps1-Δ1 (pRS403-FPS1-Δ1) were grown on YPD plates with or without 0.8 M KCl for 1–2 days at 30°C. Cell growth shown in (b) correlates well with the ability of those cells to accumulate glycerol (c) and recover cell volume (d) after osmotic treatment. For cell volume data, line thickness indicates standard deviation of data obtained with approximately 30 cells. See Methods for the details of glycerol assay and cell volume measurement.

The aquaglyceroporin Fps1 acts as a facilitator for glycerol efflux^32^,^33^. Proper Fps1 gating requires its N- and C-terminal regions and unregulated Fps1 causes sensitivity to osmotic^34^,^35^, arsenite^36^ and acetic acid stress^37^. Fps1 gating upon osmostress appears to be controlled by Hog1 via phosphorylation of Fps1^36^ and its regulator Rgc2^38^ (Fig. 3a). To examine the importance of this step, we introduced a deletion of *FPS1* (*fps1Δ*) or hyperactive Fps1 (N-terminal truncated *FPS1-Δ1*) into the synthetic osmoadaptation strain (*hog1Δ* with *PFUS1-GPD1^4A^ PFUS1-GPP2*). The presence or absence of *FPS1* did not affect the synthetic osmoadaptation where Fps1 gating cannot be regulated by Hog1, while expression of the unregulated *FPS1-Δ1* caused a strong osmosensitive phenotype (Fig. 3b). Moreover, growth of the engineered strains under osmotic condition showed a good correlation with their ability to accumulate glycerol (Fig. 3c) and recover cell volume (Fig. 3d). These results strongly suggest that Hog1-dependent regulation of Fps1 gating is dispensable for synthetic osmoadaptation although control of Fps1 is essential.

### Prevention of osmostress-induced abnormal morphology does not affect osmoadaptation

**Figure 4:**
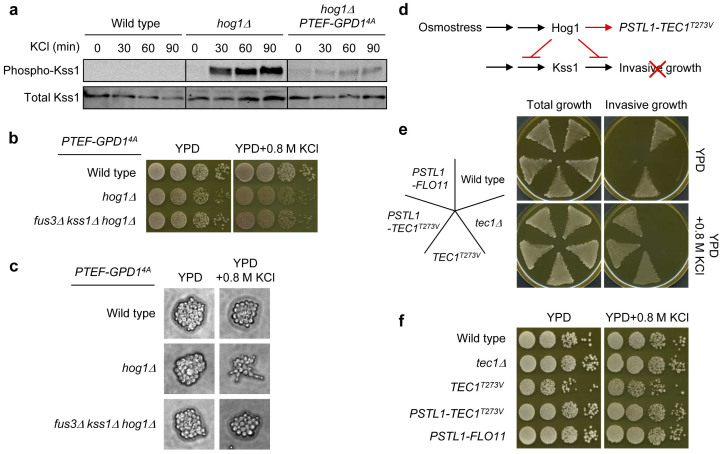
Prevention of osmostress-induced abnormal morphology does not affect osmoadaptation. (a) Constitutive expression of *GPD1^4A^* attenuates crosstalk in *hog1Δ*. Kss1 phosphorylation upon osmotic stress (0.4 M KCl) was monitored at 0, 30, 60, and 90 minutes by Western blot analysis using an anti-phospho p42/44 antibody. The protein level of Kss1 (detected using anti-Kss1 antibody) served as a loading control. Full-length blots are presented in Supplementary Figure 1. (b, c) Different cell morphology in the presence or absence of crosstalk does not affect synthetic osmoadaptation. Cells of the indicated strains carrying YIplac128-PTEF-GPD1^4A^ were grown on YPD plates with or without 0.8 M KCl for 1–2 days at 30°C (b). Cell morphology after one day was observed under the microscope (c). (d) Experimental design for forced osmotic induction of invasive growth. (e) Osmotic induction of filamentous growth (invasive growth) by expressing *TEC1^T273V^* or *FLO11* under the control of the osmoresponsive *STL1* promoter. Cells of different strains in the Σ1278b background were patched on YPD plates with or without 0.8 M KCl, grown for 1–2 days at 30°C (left), and then washed with water (right). (f) Prevention, forced osmotic induction, or constitutive induction of filamentous growth (invasive growth) does not affect osmoadaptation. Cell growth of the indicated Σ1278b strains was examined as in (b).

The mechanism how Hog1 prevents crosstalk between the Hog1 and Fus3/Kss1 MAPK pathways, which share several upstream components including Ste20, Ste50, and Ste11, has been extensively studied^39^. Since previous studies of crosstalk in *hog1Δ* cells did not consider glycerol accumulation, we examined whether overexpression of *GPD1^4A^* (*PTEF-GPD1^4A^*) affects crosstalk. In contrast to sustained activation of Kss1 in *hog1Δ* cells upon osmotic stress^40^, we found that activation of Kss1 in *hog1Δ* cells overexpressing *GPD1^4A^* was strongly attenuated (Fig. 4a). As expected, the *hog1Δ* cells overexpressing *GPD1^4A^* were able to grow well under hyperosmotic condition (Fig. 4b). Importantly, growth supported by overexpressed *GPD1^4A^* appeared to be better than that of the *hog1Δ* cells carrying *PFUS1-GPD1^4A^/PFUS1-GPP2*, which showed strong activation of Kss1 upon osmotic stress (Supplementary Fig. 1). These results suggest that prevention of strong crosstalk does not necessarily require Hog1 itself as long as *GPD1* is upregulated before subjection to osmotic stress and that glycerol accumulation and prevention of crosstalk together result in better synthetic osmoadaptation than glycerol accumulation alone.

Crosstalk in the *hog1Δ* mutant is known to cause a shmoo-like cell morphology and growth arrest^24^ and a previous study suggested that this abnormal morphology contributes to the osmosensitivity of *hog1Δ*^25^. Overexpression of *GPD1^4A^* partly suppressed the abnormal morphology of *hog1Δ* (Fig. 4c) and this suppression is probably due to attenuated crosstalk. Although blocking the crosstalk by deletion of both *FUS3* and *KSS1* genes completely prevented the abnormal morphology, it did not affect growth under hyperosmotic condition in any way (Fig. 4b, c). These results suggest that abnormal morphology itself does not affect osmoadaptation.

To verify that osmoadaptation does not require prevention of osmostress-induced abnormal morphology, we constructed yeast strains in which filamentous growth (invasive growth) is induced upon osmotic stress and examined whether it affects osmoadaptation. We engineered a Σ1278b strain, which is commonly used for the study of filamentous growth^41^, such that the cells express a constitutively stable *TEC1^T273^* gene (Tec1 is a transcription factor responsible for expression of genes required for invasive growth) under the control of a Hog1-dependent osmoresponsive *STL1* promoter (Fig. 4d). As shown in Figure 4e, the wild-type and *tec1Δ* cells did not invade into the osmotic agar plate, while the cells carrying *TEC1^T273V^* (constitutive expression) or *PSTL1-TEC1^T273V^* were able to invade into the osmotic stress agar plate. Importantly, all of these strains grew normally under osmotic stress (Fig. 4f). Moreover, yeast cells that upregulated *FLO11* (mucin protein responsible for invasive growth) under the control of *STL1* promoter showed almost the same growth patterns as cells carrying *PSTL1-TEC1^T273V^* (Fig. 4e, f). These results indicate that prevention, forced osmotic induction, or constitutive induction of invasive growth does not affect osmoadaptation. Therefore, prevention of abnormal morphology by Hog1 appears to be dispensable for osmoadaptation.

## Discussion

In this report, we reconstituted osmoadaptation in *hog1Δ* cells by rewiring osmostress signalling through the MAPK network. To our knowledge, this is the first time that yeast osmoadaptation was synthetically mediated through a different MAPK pathway. In addition, our approach made it possible to examine each Hog1-dependent osmostress-induced gene in the *hog1Δ* mutant background in which all of the Hog1-dependent induction is eliminated. Although our *hog1Δ* strain showing synthetic osmoadaptation (with *PFUS1-GPD1/GPP2*) does not have the same capability of establishing osmoresistance as wild type, our data indicate that one of the essential roles of Hog1 for osmoadaptation is osmotic induction of two glycerol biosynthesis genes, *GPD1* and *GPP2* (Fig. 2). Since Hog1 induces many additional genes, osmotic upregulation of each Hog1-dependent gene may improve the synthetic osmoadaptation. In addition to analysis of systems level properties employing different mutants as system perturbations^42^,^43^, such a synthetic approach may also contribute to a quantitative understanding of role of a given Hog1-dependent gene or mechanism in osmoadaptation. However, many of the Hog1-dependent genes do not seem to affect osmoadaptation when deleted individually as suggested by data in Figure 1c, Table S1 and previous reports^17^,^23^. Hence, although the physiological reasons why osmotic stress causes upregulation of so many genes remain unclear, this study suggests that the number of essential genes regulated by MAPKs for a specific response or adaptation may be significantly smaller than anticipated.

Nuclear translocation of Hog1 upon osmotic stress^44^ and the induction of osmoresponsive genes had long been assumed to be necessary for coping with hyperosmotic stress. Contrary to this assumption, Westfall *et al*. reported that yeast cells lacking Nmd5 (importin-β homologue) required for Hog1 nuclear import or cells in which Hog1 is tethered to the plasma membrane can adapt to hyperosmotic conditions without Hog1-dependent induction of the osmoresponsive genes^17^. Although our approach is completely different from theirs, these two genetic engineering approaches may suggest that yeast cells can overcome hyperosmotic stress if one or two critical roles of Hog1 are maintained (Hog1 activity and prevention of crosstalk in Westfall's strain; osmotic induction of *GPD1* and *GPP2* in the strains developed here). This idea is further supported by the fact that overexpression of *GPD1^4A^*, which causes both crosstalk attenuation and glycerol accumulation, results in improved synthetic osmoadaptation.

Our observation suggests also that Hog1 is dispensable for regulation of Fps1 under hyperosmotic condition although proper Fps1 gating (closing) itself is essential (Fig. 3b–d). This result extends a previous finding that even *hog1Δ* cells can reduce glycerol transport activity upon hyperosmotic shock^33^. While this manuscript was in preparation, Levin and coworkers showed that Hog1 closes Fps1 by phosphorylating and displacing the Rgc2 regulator from the C-terminal domain of Fps1^38^. Moreover, they concluded that Hog1 uses the N-terminal domain of Fps1 as a platform to evict Rgc2 from Fps1. However, since deletion of *HOG1* does not completely abolish *in vivo* phosphorylation of Fps1^36^ and Rgc2^45^, other kinases may also be involved in Fps1 gating. At least, we did not observe an osmosensitive phenotype caused by deletion of other MAPK genes (*FUS3*, *KSS1*, and *SLT2*/*MPK1*) in *hog1Δ* cells overexpressing *GPD1^4A^* (Fig. 4b and data not shown), suggesting that Fps1 might be regulated also in a MAPK-independent manner. Changes of turgor pressure or cell volume upon osmotic stress may contribute, probably transiently, to regulation of Fps1 even without Hog1 and/or other MAPKs.

Abnormal cell morphology following osmostress is observed in *hog1Δ* cells even when *GPD1^4A^* is overexpressed, while it is completely prevented by deletion of *FUS3* and *KSS1* (Fig. 4c). Although a previous study showed that prevention of crosstalk by deletion of *KSS1* partially suppresses osmosensitivity^25^, our results indicate that *hog1Δ* cells with abnormal or normal morphology are capable of acquiring osmoresistance as long as *GPD1* is upregulated (Fig. 4b). Therefore, our results suggest that prevention of crosstalk indeed suppresses osmosensitivity, but prevention of osmostress-induced abnormal morphology itself is dispensable. In the Σ1278b strain background, Hog1 acts as a central negative regulator of morphological developments^46^,^47^ including fluffy colony morphology, invasive growth, and pseudohyphal development, which all are stimulated by the Kss1 MAPK. We demonstrated using a Σ1278b strain background that forced osmotic induction of invasive growth does not impair osmoadaptation (Fig. 4e). Hence, our results strongly suggest that inhibitory regulation of morphological developments is not required for proper osmoadaptation. In addition to investigating how the crosstalk between MAPK pathways is prevented, it would be interesting to understand whether there is a trade-off between osmoadaptation and morphological developments.

Genetic analysis of signal transduction systems by gene deletion or overexpression and phenotypic characterization is well-known to be prone to incorrect interpretation because of compensatory effects, altered protein complex formation and altered pathway crosstalk. Here we present synthetic osmoadaptation in a Hog1-independent manner by using a complementary approach, which provided novel insights into yeast osmoadaptation. Although we focused on glycerol biosynthesis genes, Fps1 regulation, and crosstalk inhibition, Hog1 plays many further roles such as controls of cell cycle^8^ and translation^48^ and there are many candidate substrates of Hog1^49^. We expect that reconstitution of each role one by one may lead to better understanding of the truly essential Hog1's roles. Moreover, it would be interesting to reconstitute osmoadaptation using orthogonal control systems such as light and hormones. This kind of effort may contribute to creating novel synthetic signalling pathways with predictable behaviours useful for future applications in medicine and biotechnology.

## Methods

### Yeast media and growth conditions

Standard media, SC (synthetic complete: 2% glucose, 0.67% yeast nitrogen base without amino acids, and supplemented with amino acids to satisfy nutritional requirements) and YPD (1% yeast extract, 2% peptone, and 2% glucose), were used for yeast cultivation and selection of transformants. For growth assays to examine osmosensitivity, cells were pregrown overnight on YPD plates, resuspended in water to OD_600_ = 0.1, and 5 μl of a 10-fold dilution series were spotted onto YPD plates with or without KCl. Cell growth or morphology was monitored after 1–2 days culture at 30°C.

### Yeast strains and plasmids

Yeast strains, plasmids, and primers used in this study are listed in Table S2, Table S3, and Table S4, respectively. Yeast transformation and gene deletion were performed as described previously^50^. Plasmids for *GPD1* and *GPP2* expression under the control of *FUS1* or *TEF* promoter were constructed in the YIp352 (*URA3* marker) or YIplac128 (*LEU2* marker) backbones. A *GPD1* fragment was obtained from pCM190HH-GPD1 (Markus Tamás). *GPP2*, *PFUS1*, and *PTEF* fragments were obtained by PCR using yeast genomic DNA or plasmid (pYM-N18 for *PTEF*) as a template. *GPD1^4A^* and *TEC1^T273V^* mutations were generated by overlap extension PCR-mediated mutagenesis. A plasmid for *FPS1-Δ1* expression was constructed by insertion of the *FPS1-Δ1* fragment derived from YIpURA3-FPS1-Δ1 (Markus Tamás) into pRS403 (*HIS3* marker). *PSTL1-TEC1^T273V^* and *PSTL1-FLO11* strains were constructed by replacement of their original promoter regions with a *LEU2-PSTL1* cassette.

### Measurement of intracellular glycerol

Cells were grown to mid-log phase in 30 ml of YPD liquid medium. KCl was added to the medium to a final concentration of 0.8 M, and 1 ml aliquots were withdrawn after 0, 2, 4, and 6 hours. Cells were harvested and resuspended in 1 ml of water and boiled at 100°C for 10 min, and supernatants were stored at −20°C. OD_600_ was determined at all-time points. Glycerol concentration was determined using a commercial kit (Roche Applied Science). Reaction was scaled down 12 times to a final reaction volume of 250 μl. Measurements were performed in a 96-well plate using a Polar Star Omega plate reader (BMG Labtech). The mean value of glycerol/OD_600_ ± S.D. (n = 3) was plotted versus time.

### Single cell analysis of cell volume

Single cell analysis of cell volume upon osmotic stress (0.8 M KCl) was performed using a microfluidic system with three inlet channels as described previously^29^,^30^. Images of approximately 30 cells were taken sequentially every 30 sec for 5 min, every 1 min for 5 min, every 10 min for 20 min, and every 30 min for 90 min, thus yielding a total experiment period of 120 min. The images were analyzed using CellStress software^51^.

### Western blot

Cells were grown to mid-log phase in 30 ml of YPD liquid medium. KCl was added to the medium to a final concentration of 0.4 M, and 1 ml aliquots were withdrawn at times 0, 30, 60, and 90 min. Cells were resuspended in standard SDS loading buffer, boiled for 10 min, and sedimented at 13,000 × *g* at 4°C for 10 min to obtain protein extracts. Protein extracts (45 μg) were examined for Western blot analysis using anti-phospho-p44/42 MAPK antibody (Cell Signaling Technology) or anti-Kss1 antibody (y-50, Santa Cruz Biotechnology) as described previously^30^.

## Author Contributions

K.F. designed and supervised the study; R.B., T.F. and K.F. performed the experimental work; R.B., S.H. and K.F. analysed results and wrote the manuscript.

## Supplementary Material

Supplementary Information Supplementary Information
