# Supplementary material for: Rewiring yeast osmostress signalling through the MAPK network reveals essential and non-essential roles of Hog1 in osmoadaptation
Source: Sci Rep. 2014 Apr 15;4:4697. doi: 10.1038/srep04697 (PMC3986706; doi:10.1038/srep04697)
Supplement: Supplementary Information [file srep04697-s1.doc]

**Supplementary Information**

**Rewiring yeast osmostress signalling through the MAPK network reveals essential and non-essential roles of Hog1 in osmoadaptation**

**Roja Babazadeh, Takako Furukawa, Stefan Hohmann & Kentaro Furukawa**

Department of Chemistry and Molecular Biology, University of Gothenburg, Gothenburg, Sweden.

Correspondence and requests for materials should be addressed to

S.H. (stefan.hohmann@gu.se) or K.F. (kentaro.furukawa@cmb.gu.se)


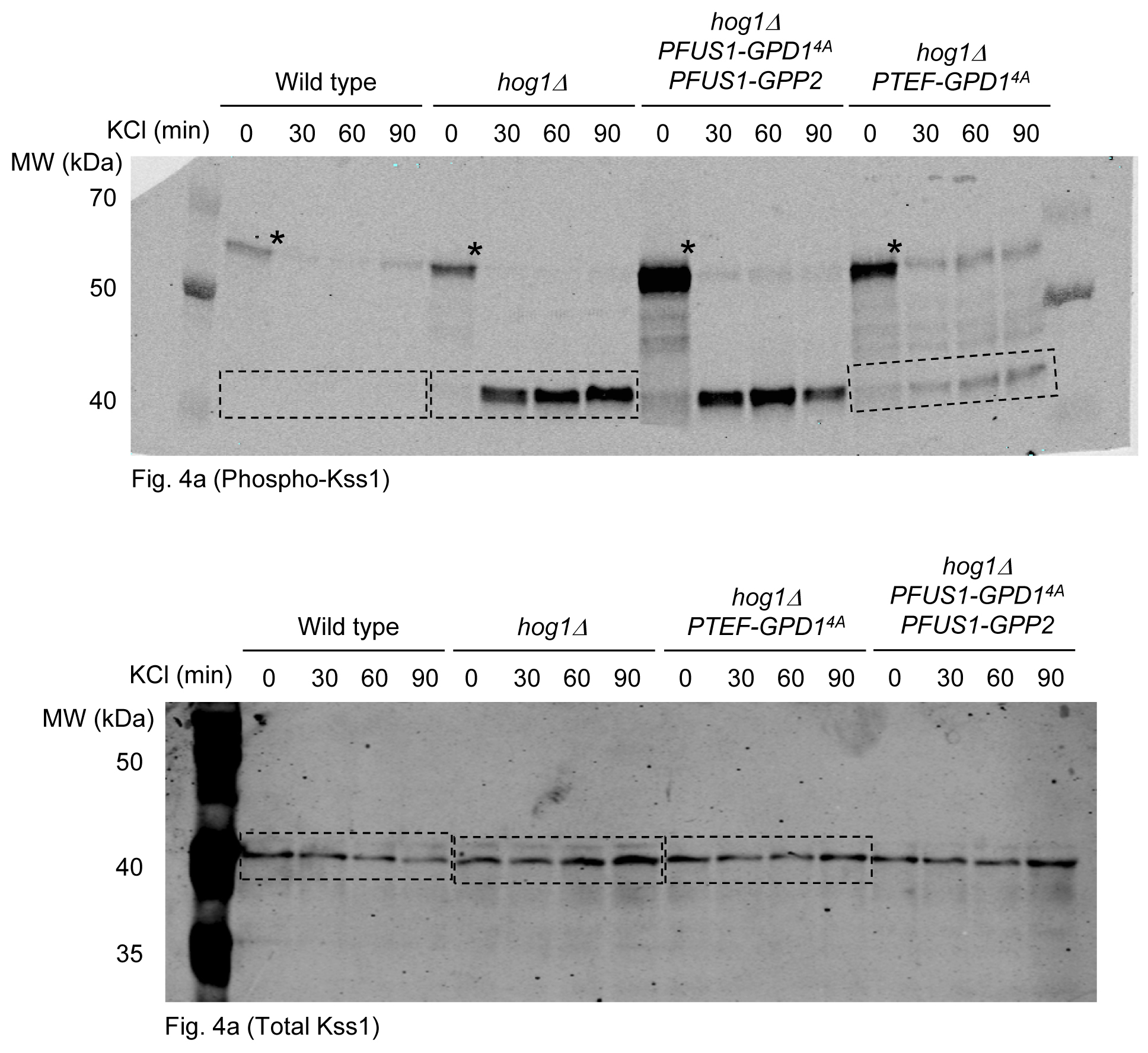


**Figure S1 | Full-length blot images for Figure 4a.**

Asterisk bands (*) are phosphorylated signals of the Mpk1/Slt2 MAPK (known to become unphosphorylated upon osmotic shock).

**Table S1 | Growth of yeast strains lacking genes whose osmotic upregulation strongly depends on Hog1**

Systematic name Gene name Description of gene product Growth on 0.8 M KCl

*YMR169C* *ALD3* Cytoplasmic aldehyde dehydrogenase Yes

*YFL014W* *HSP12* Heat shock protein Yes

*YHR087W* *RTC3* Protein of unknown function involved in RNA metabolism Yes

*YGR043C* *NQM1* Transaldolase of unknown function Yes

*YML128C* *MSC1* Protein of unknown function Yes

*YDL223C* *HBT1* Shmoo tip protein (substrate of Hub1p ubiquitin-like protein) Yes

*YGR248W* *SOL4* 6-phosphogluconolactonase Yes

*YHR139C* *SPS100* Sporulation specific protein required for spore wall maturation Yes

*YMR170C* *ALD2* Cytoplasmic aldehyde dehydrogenase Yes

*YGR088W* *CTT1* Cytosolic catalase T Yes

***YDL022W* *GPD1* NAD-dependent glycerol-3-phosphate dehydrogenase No**

*YDR536W* *STL1* Glycerol proton symporter of the plasma membrane Yes

*YER062C* *GPP2* Glycerol-3-phosphatase involved in glycerol biosynthesis Yes

*YER150W* *SPI1* GPI-anchored cell wall protein involved in weak acid resistance Yes

*YOR374W* *ALD4* Mitochondrial aldehyde dehydrogenase Yes

*YDR074W* *TPS2* Phosphatase subunit of the trehalose-6-P phosphatase complex Yes

*YML070W* *DAK1* Dihydroxyacetone kinase Yes

*YLR178C* *TFS1* Phosphatidylethanolamine-binding protein family member Yes

*YKL151C YKL151C* NADHX dehydratase Yes

*YHR137W* *ARO9* Aromatic aminotransferase II Yes

*YAL061W* *BDH2* Putative medium-chain alcohol dehydrogenase Yes

*YHL021C* *AIM17* Protein of unknown function Yes

*YHR104W* *GRE3* Aldose reductase Yes

*YDR504C* *SPG3* Protein required for survival at high temperature Yes

*YMR090W* *YMR090W* Putative protein of unknown function Yes

*YML100W* *TSL1* Subunit of trehalose 6-phosphate synthase/phosphatase complex Yes

*YGR243W* *FMP43* Highly conserved subunit of mitochondrial pyruvate carrier Yes

*YMR174C* *PAI3* Cytoplasmic proteinase A (Pep4) inhibitor Yes

*YPL223C* *GRE1* Hydrophilin essential in dessication-rehydration process Yes

*YOL151W* *GRE2* D-lactaldehyde dehydrogenase Yes

*YML004C* *GLO1* Monomeric glyoxalase I Yes

*YLL023C* *POM33* Transmembrane nucleoporin Yes

*YBR117C* *TKL2* Transketolase Yes

*YCL064C* *CHA1* Catabolic L-serine (L-threonine) deaminase Yes

*YKL096W* *CWP1* Cell wall mannoprotein Yes

*YML131W* *YML131W* Protein of unknown function Yes

*YGR086C* *PIL1* Primary protein component of eisosomes Yes

*YMR175W* *SIP18* Phospholipid-binding hydrophilin Yes

*YLL026W* *HSP104* Disaggregase (heat shock protein) Yes

*YOL084W* *PHM7* Protein of unknown function Yes

*YIL136W* *OM45* Mitochondrial outer membrane protein of unknown function Yes

*YIL053W* *GPP1* Glycerol-3-phosphatase Yes

*YOR317W* *FAA1* Long chain fatty acyl-CoA synthetase Yes

*YJL108C* *PRM10* Pheromone-regulated protein Yes

*YJL164C* *TPK1* cAMP-dependent protein kinase catalytic subunit Yes

YGR130C *YGR130C* Component of the eisosome with unknown function Yes

Genes are sorted according to the fold-induction after osmotic shock in wild type. The fold induction of all these genes is diminished by more than 75% by deletion of *HOG1*. See the following paper for the details.

Rep, M., Krantz, M., Thevelein, J. M. & Hohmann, S. The transcriptional response of *Saccharomyces cerevisiae* to osmotic shock. Hot1p and Msn2p/Msn4p are required for the induction of subsets of high osmolarity glycerol pathway-dependent genes. *J. Biol. Chem.* **275,** 8290–8300 (2000).

**Table S2 | Yeast strains used in this study**

Strain Genotype Source

BY4741 *MATa his3Δ1 leu2Δ0 met15Δ0 ura3Δ0*  EUROSCARF

BY4741 *xxxΔ* BY4741 *xxx::kanMX* (used for Table S1) EUROSCARF

W303-1A *MATa leu2-3,112 trp1-1 his3-11,15 ura3-1 can1-100 ade2-1* Lab collection

YSH404 W303-1A *hog1::TRP1* Lab collection

YSH1425 W303-1A *fus3::kanMX kss1::kanMX hog1::TRP1* Lab collection

10560-6B *MATα leu2::hisG trp1::hisG his3::hisG ura3-52* Lab collection

YSH1888 10560-6B *tec1::kanMX* Lab collection

YSH2451 W303-1A *ura3-1::YIp352* This study

YSH2452 W303-1A *ura3-1::YIp352-PFUS1-GPD1* This study

YSH2454 W303-1A *hog1::TRP1* *ura3-1::YIp352*  This study

YSH2455 W303-1A *hog1::TRP1* *PFUS1::YIp352-PFUS1-GPD1* This study

YSH2457 W303-1A *fus3::kanMX kss1::kanMX hog1::TRP1* *ura3-1::YIp352*  This study

YSH2458 W303-1A *fus3::kanMX kss1::kanMX hog1::TRP1* *PFUS1::YIp352-PFUS1-GPD1* This study

YSH2761 W303-1A *hog1::TRP1* *PFUS1::YIp352-PFUS1-GPP2* This study

YSH2766 W303-1A *hog1::TRP1* *PFUS1::YIp352-PFUS1-GPP2 leu2-3,112::YIplac128-PFUS1-GPD1* This study

YSH2767 W303-1A *hog1::TRP1* *PFUS1::YIp352-PFUS1-GPP2 leu2-3,112::YIplac128-PFUS1-GPD1^4A^* This study

YSH2777 W303-1A *hog1::TRP1* *PFUS1::YIp352-PFUS1-GPP2 leu2-3,112::YIplac128-PFUS1-GPD1^4A^* This study

*his3-11,15::pRS403*

YSH2803 W303-1A *fps1::HIS3 hog1::TRP1* *PFUS1::YIp352-PFUS1-GPP2 leu2-3,112::YIplac128-PFUS1-GPD1^4A^* This study

YSH2804 W303-1A *fps1::HIS3 hog1::TRP1* *PFUS1::YIp352-PFUS1-GPP2 leu2-3,112::YIplac128-PFUS1-GPD1^4A^* This study

*his3-11,15::pRS403-fps1-Δ1*

YSH2805 W303-1A *leu2-3,112::YIplac128-PTEF-GPD1^4A^* This study

YSH2806 W303-1A *hog1::TRP1 leu2-3,112::YIplac128-PTEF-GPD1^4A^*  This study

YSH2807 W303-1A *fus3::kanMX kss1::kanMX hog1::TRP1 leu2-3,112::YIplac128-PTEF-GPD1^4A^*  This study

YSH2221 10560-6B *TEC1^T273V^* This study

YSH2499 10560-6B *flo11::PSTL1-FLO11-LEU2* This study

YSH2503 10560-6B *tec1::PSTL1-TEC1^T273V^-LEU2*  This study

**Table S3 | Plasmids used in this study**

Plasmid Description Source

YIp352 *URA3* 1

YIplac128 *LEU2* 2

pRS403 *HIS3* 3

pYM-N18 *kanMX PTEF* 4

pCM190HH-GPD1 *2μ URA3 PtetOFF-His6-HA-GPD1* 5

YIp352-PFUS1-GPD1 *URA3 PFUS1-GPD1* This study

YIp352-PFUS1-GPP2 *URA3 PFUS1-GPP2* This study

YIplac128-PFUS1-GPD1 *LEU2 PFUS1-GPD1* This study

YIplac128-PFUS1-GPD1^4A^ *LEU2 PFUS1-GPD1(S23/24/25/27A)* This study

YIplac128-PTEF-GPD1^4A^ *LEU2 PTEF-GPD1(S23/24/25/27A)* This study

pRS403-FPS1-Δ1 *HIS3 PFPS1-FPS1-Δ1* This study

**References**

1. Hill, J.E., Myers, A.M., Koerner, T.J. & Tzagoloff, A. Yeast/*E. coli* shuttle vectors with multiple unique restriction sites. *Yeast* **2,** 163–167 (1986).
2. Gietz, R.D. & Sugino, A. New yeast-*Escherichia coli* shuttle vectors constructed with in vitro mutagenized yeast genes lacking six-base pair restriction sites. *Gene* **74,** 527–534 (1988).
3. Sikorski, R.S. & Hieter, P. A system of shuttle vectors and yeast host strains designed for efficient manipulation of DNA in *Saccharomyces cerevisiae*. *Genetics* **122,** 19–27 (1989).
4. Janke, C. *et al*. A versatile toolbox for PCR-based tagging of yeast genes: new fluorescent proteins, more markers and promoter substitution cassettes. *Yeast* **21,** 947–62 (2004).
5. Fillinger, S. *et al*. Molecular and physiological characterization of the NAD-dependent glycerol 3-phosphate dehydrogenase in the filamentous fungus *Aspergillus nidulans*. *Mol. Microbiol.* **39,** 145–157 (2001).

**Table S4 | Primers used in this study**

Primer Sequence (5’ to 3’)

**For construction of *PFUS1-GPD1***

P-PFUS1(EcoRI)F CCAGAACCGCGAATTCACGATGATTCAGTTC

P-PFUS1-MetGPD1(BamHI)R CAGCAGCAGAGGATCCCATTTTGATTTTCAGAAAC

**For construction of *PFUS1-GPP2***

P-GPP2(BamHI)F ATTCGGAATGGGATCCACTACTAAACCTCTATC

P-GPP2(NotI)R ATGTTCGATTGCGGCCGCTTACCATTTCAACAGATCG

**For *GPD1^4A^* mutation**

P-GPD1(4A)F GAATGCTGGTAGAAAGAGAAGTGCCGCAGCTGTTGCTTTGAAGGCTGCCGAAAAGCC

P-GPD1(4A)R GGCTTTTCGGCAGCCTTCAAAGCAACAGCTGCGGCACTTCTCTTTCTACCAGCATTC

**For construction of *PTEF-GPD1^4A^***

P-PTEF(+EcoRI)F ATCCAGTGTCGAATTCGAGCTCATAGCTTC

P-PTEF(+ATG+BamHI)R GGGGAACCACGGATCCCATAAAACTTAGATTAGATTGC

**For *TEC1^T273V^* mutation**

P-TEC1-T273V(F) GTACATTCAAAACTGCTTGTACCAATCACTGCTTCCAACG

P-TEC1-T273V(R) CGTTGGAAGCAGTGATTGGTACAAGCAGTTTTGAATGTAC

**For construction of *PSTL1-TEC1^T273V^***

P-LEU2-PSTL1-TEC1(F) CACCTGTAGCTACCATCAGCAATCCATGGTGCTGGAGTTTCTCTCGATGGAACTGTGGGAATACTCAGGTATCG

P-LEU2-PSTL1-TEC1(R) TCTATATTTCTAGAATTATCCTTGCCAAAGTCGTCTTCTTTAAGACTCATGGGGTCTAAAACTTTCTATGTTCTATTTTTC

**For construction of *PSTL1-FLO11***

P-LEU2-PSTL1-FLO11(F) GGGCAGTTTTATTTACCTTAACAAATATGTTCAAGCATTTACGTTACTGCAACTGTGGGAATACTCAGGTATCG

P-LEU2-PSTL1-FLO11(R) TTAAATAGAAGCGAAAGGACCAAATAAGCGAGTAGAAATGGTCTTTGCATGGGGTCTAAAACTTTCTATGTTCTATTTTTC

Primers used for gene deletion are not shown here.
